# Crosstalk between Adipose Tissue and Hepatic Mitochondria in the Development of the Inflammation and Liver Injury during Ageing in High-Fat Diet Fed Rats

**DOI:** 10.3390/ijms24032967

**Published:** 2023-02-03

**Authors:** Gina Cavaliere, Angela Catapano, Giovanna Trinchese, Fabiano Cimmino, Ciro Menale, Lidia Petrella, Maria Pina Mollica

**Affiliations:** 1 Department of Pharmaceutical Sciences, University of Perugia, 06126 Perugia, Italy; 2 Centro Servizi Metrologici e Tecnologici Avanzati (CeSMA), Complesso Universitario di Monte Sant’Angelo, Via Cinthia 21, 80126 Naples, Italy; 3 Department of Biology, University of Naples Federico II, 80126 Naples, Italy; 4 Department of Clinical Medicine and Surgery, University of Naples Federico II, 80131 Naples, Italy; 5 Task Force on Microbiome Studies, University of Naples Federico II, 80138 Naples, Italy

**Keywords:** inflammation, adipose tissue, liver diseases, mitochondrial function, adipocyte size

## Abstract

Obesity is considered an epidemic disorder, due to an imbalance between energy consumption and metabolizable energy intake. This balance is increasingly disrupted during normal aging processes due to the progressive impairment of mechanisms that normally control energy homeostasis. Obesity is triggered by an excessive lipid depots but reflects systemic inflammation along with large adipocytes secreting proinflammatory adipokines, an increase of the free fatty acids levels in the bloodstream, and ectopic lipid accumulation. Hepatic fat accumulation is the most common cause of chronic liver disease, characterized by mitochondrial dysfunction with a consequent impaired fat metabolism and increased oxidative stress. Therefore, mitochondrial dysfunction is associated to hepatic lipid accumulation and related complications. In this study, we assessed the crosstalk between adipose tissue and liver, analyzing the time-course of changes in hepatic mitochondrial fatty acid oxidation capacity versus fatty acid storage, focusing on the contribution of adipose tissue inflammation to hepatic lipid accumulation, using a rodent model of high fat diet-induced obesity. Our results demonstrate that both high-fat diet-induced obesity and aging induce dysregulation of adipose tissue function and similar metabolic alterations mediated by mitochondrial function impairment and altered inflammatory profile. The high fat diet-induced obesity anticipates and exacerbates liver mitochondrial dysfunction that occurs with aging processes.

## 1. Introduction

In industrialized countries, there has been an alarming increase in obesity incidence in recent decades, reaching epidemic proportions and increasing the prevalence of metabolic disorders [1]. This condition is caused by a body imbalance between energy consumption and metabolizable energy (ME) intake and it is mainly related to a sedentary lifestyle and high consumption of fat-rich food. Several studies have highlighted a tight association between obesity and the normal aging process [2]. In fact, a slow but progressive increase in adiposity occurs throughout most of adulthood [3,4], due to the progressive impairment of mechanisms that normally control energy homeostasis [5]. Several evidence has shown that the incidence of obesity is increasing in middle-aged people, and may accelerate the aging process [6]. Specifically, the adipose mass is determined by the energetic balance between net fat storage in adipocytes and total fat oxidation [7]. This balance is increasingly disrupted during aging, because the capacity of tissues to oxidize fat gradually decreases. Several studies have provided evidence that obesity reflects a generalized pro-inflammatory state with increased inflammatory markers [8,9]. It is clear that tumor necrosis factor-α (TNF-α) and various other inflammatory mediators are overexpressed in adipose tissue in experimental mouse models of obesity and humans [10,11]. Further, the adipocyte size plays a key role in the secretion of pro-inflammatory adipokines. In particular, the fractionation of adipocytes according to cell size revealed that very large adipocytes show a markedly increased production of inflammatory markers [12]. The high production of these adipokines is increasingly regarded as important in the development of obesity-linked diseases and metabolic syndrome. Different studies have shown that adipose tissue dysfunction causes an increased serum level of free fatty acids and lipids with subsequent lipotoxicity [13], and ectopic lipids accumulation. Aging also promotes fat redistribution outside normal adipose tissue reservoirs, with lipid accumulation occurring not only in visceral depots, but also in other tissues [14,15], causing a higher risk of cardiometabolic disorders [16]. In particular, ectopic hepatic fat accumulation, responsible for fatty liver disease, is a widespread metabolic disorder and the most common cause of chronic liver disease in western countries, and has thus become an important public health problem. This is the manifestation of an imbalance between lipid influx and removal mechanisms [17] occurring when the availability of glucose and fatty acids exceeds both the capacity of adipose tissue to store energy and the demand of hepatocytes for adenosine triphosphate (ATP) [18]. Hepatic injury takes place when the capacity of the hepatocyte to cope with an increased level of circulating fatty acids is exceeded. It can also happen during normal aging processes, related to ectopic lipid accumulation due to the progressive deterioration of organ function. Mitochondria are cellular organelles that play a central role in the oxidation of fatty acids, so mitochondrial dysfunction is an important component in the pathophysiology of hepatic lipid accumulation and related complications [19].

Several studies have shown that aging is associated with the progressive loss of mitochondrial function in various tissues [20,21]. Therefore, mitochondrial dysfunction is known to be associated with cellular aging and senescence. To date, the pathogenesis of fatty liver disease has not been completely elucidated, but might be partly related to dysregulation of cross-talk between liver and adipose tissue [22] and the normal aging process could play an important role. In the present study, we determined the time-course of changes in mitochondrial hepatic fatty acid oxidation capacity versus fatty acid storage, analyzing the contribution of adipose tissue inflammation to hepatic lipid accumulation, using a rodent model of obesity induced by a high fat diet (HFD). In particular, we aimed to assess the crosstalk between visceral adipose tissue inflammation and parameters of liver mitochondrial function and to follow its temporal trend, in order to provide new insights into the putative mechanisms involved in the development of liver disease associated with inflammatory processes trigger.

## 2. Results

### 2.1. Body Composition and Energy Balance

**Figure 1:**
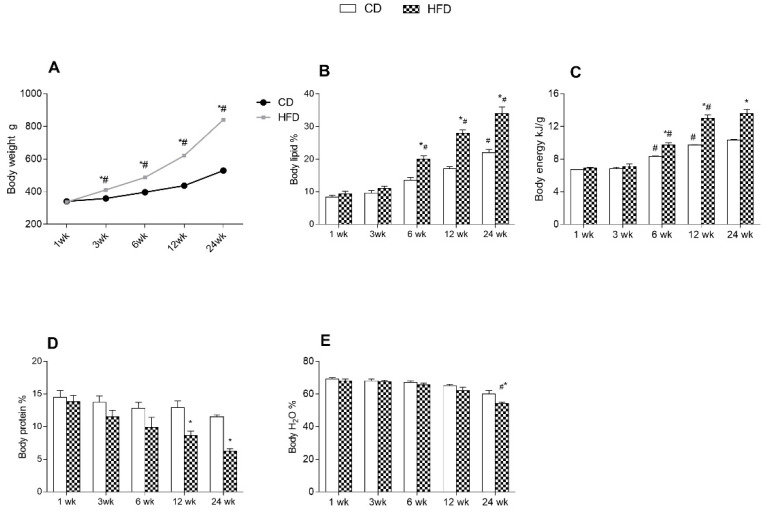
Effects of high-fat diet and aging on body weight and body composition. All the parameters were measured throughout the experimental period (1–24 weeks): (**A**) body weight; (**B**) body lipid; (**C**) body energy; (**D**) body protein and (**E**) body water are shown. Data are indicated as means ± SEM from n = 6 animals/group. # Significantly different compared to previous time point, *p* < 0.05; * significantly different compared to CD group, *p* < 0.05.

We first analyzed the effects of an HFD and aging on body composition and energy balance at different times: 1, 3, 6, 12, and 24 weeks of treatment. The average body weight of all groups of rats, measured throughout the experimental period, is given in Figure 1A. The graph clearly shows a significant increase in body weight throughout the experimental period in HFD-fed rats compared to the control group (CD), fed a standard diet. Both HFD and CD groups displayed a progressive increase in body weight, body lipid, and body energy content with age. All parameters were higher in the HFD than in the CD group at each time point, but the differences were statistically significant from about 6 weeks onward (Figure 1A–C). Evaluation of the percentage of body water and body protein showed a progressive decrease in both groups with age. This reduction is more pronounced in HFD rats than in the other group from the 12 week (Figure 1D,E).

**Figure 2:**
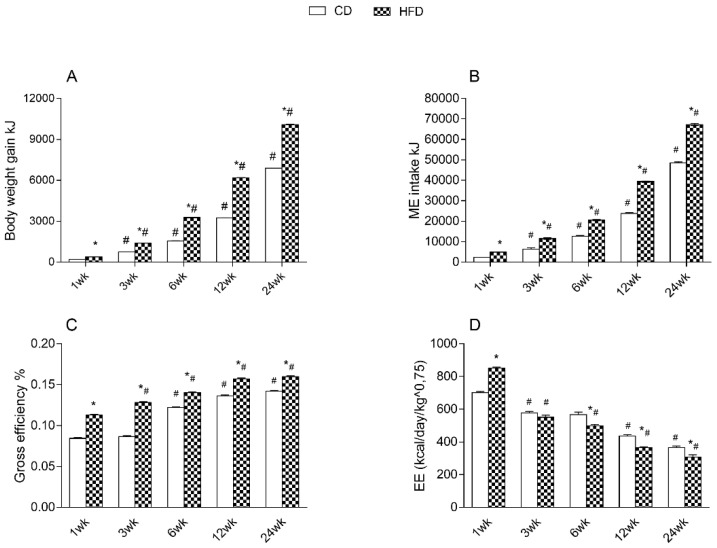
Effects of high-fat diet and aging on energy balance. All the parameters were measured throughout the experimental period (1–24 weeks): (**A**) body weight gain; (**B**) metabolizable energy (ME) intake; (**C**) gross efficiency; (**D**) energy expenditure (EE) are shown. Data are indicated as means ± SEM from n = 6 animals/group. # Significantly different compared to previous time point, *p* < 0.05; * significantly different compared to CD group, *p* < 0.05.

To obtain further insight into the changes in body composition, we assessed the main components of energy balance. As reported in Figure 2A–C, body weight gain, ME, and gross efficiency were progressively augmented with age in both animal groups, at every time point, with significantly higher values in the HFD compared to CD rats. On the other hand, we found a reduction in the energy expenditure (EE) with age in both groups of rats at each time point, with significantly lower values in HFD rats compared to control rats from 6 week (Figure 2D).

### 2.2. Serum Parameters

**Figure 3:**
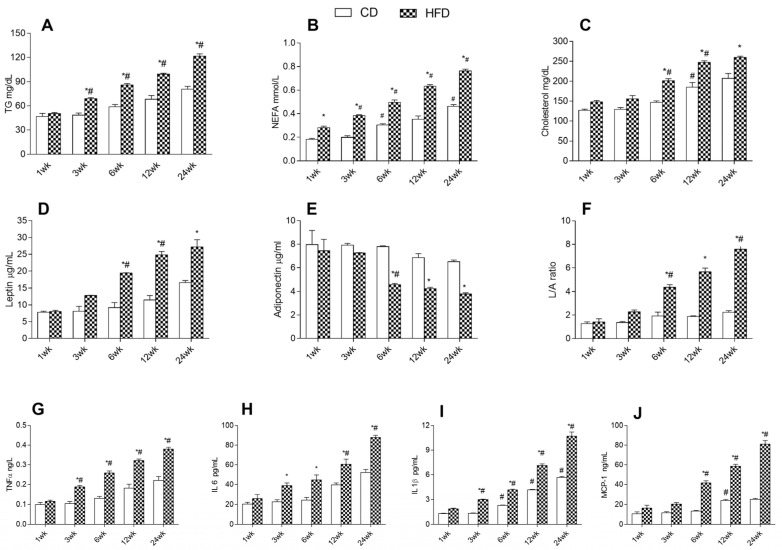
Effects of HFD and aging on serum parameters. All the parameters were measured throughout the experimental period (1–24 weeks): (**A**) triglycerides (TG); (**B**) not esterified fatty acids (NEFA); (**C**) cholesterol; (**D**) leptin; (**E**) adiponectin; (**F**) leptin/adiponectin (L/A) ratio; (**G**) tumor necrosis factors-α (TNF-α); (**H**) interleukin-6 (IL-6); (**I**) interleukin-1 (IL-1); (**J**) monocyte chemoattractant protein-1 (MCP-1) are shown. Data are indicated as means ± SEM from n = 6 animals/group. # Significantly different compared to previous time point, *p* < 0.05; * significantly different compared to CD group, *p* < 0.05.

We then focused on the effects of an HFD and aging on serum levels of dyslipidemia indicators, adipokines, and pro-inflammatory cytokines, which are associated with obesity-related diseases. The HFD group showed higher triglycerides (TG), not esterified fatty acids (NEFA), cholesterol, and leptin levels than the controls, which progressively increased with age at each time point (Figure 3A–D). In contrast, adiponectin levels diminished progressively with age in HFD starting at 6 weeks, showing lower values compared to the CD group at each time point (Figure 3E). In addition, in the HFD group, the leptin/adiponectin ratio shows values significantly increased with age from 6 weeks onwards and higher than the CD group at each time point (Figure 3F). In the CD group, no significant differences between time points were observed in TG, leptin, adiponectin levels, and in L/A ratio, although a progressive increase with age was noted; while NEFA and cholesterol levels were significantly increased starting at 6 and 12 weeks, respectively. The HFD group exhibited levels of TNF-α, interleukin-6 (IL-6), interleukin-1 β (IL-1 β), and monocyte chemoattractant protein-1 (MCP-1) progressively and significantly rising with age at each time point. CD rats displayed lower inflammatory cytokines compared to the HFD group and progressively increased with age, showing higher variations from 6 and 12 weeks onward (Figure 3G–L).

### 2.3. Adipokine Release from Adipocytes

**Figure 4:**
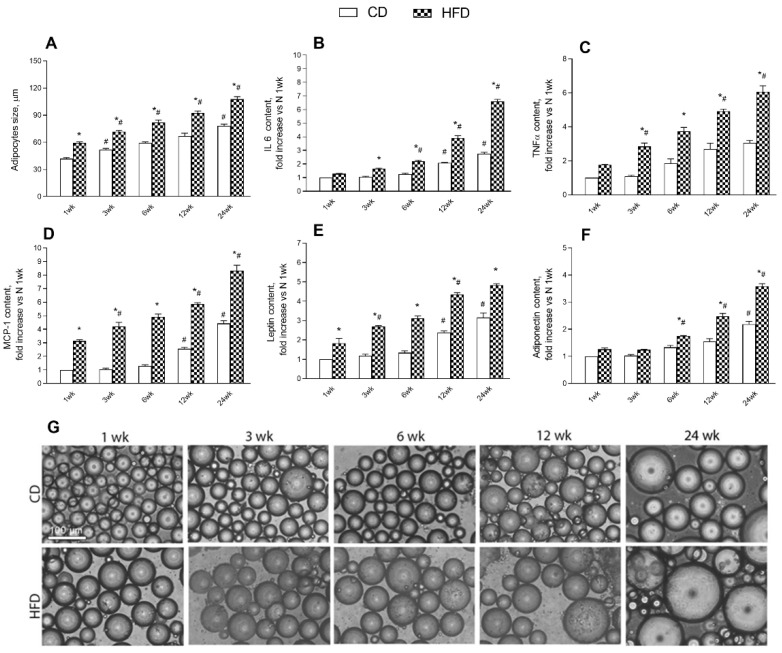
Effects of high-fat diet and aging on adipocyte size and cytokines release from adipocytes. All the parameters were measured throughout the experimental period (1–24 weeks): (**A**) adipocytes size; (**B**) interleukin-6 (IL-6); (**C**) tumor necrosis factors-α (TNF-α); (**D**) monocyte chemoattractant protein-1 (MCP-1); (**E**) leptin and (**F**) adiponectin are shown. (**G**) Representative fields of isolated adipocytes are reported for each condition and time point (scale bar = 100 μm). Data are indicated as means ± SEM from n = 6 animals/group. # Significantly different compared to previous time point, *p* < 0.05; * significantly different compared to CD group, *p* < 0.05.

In order to better investigate the contribution of a HFD and aging to metabolic characterization and inflammatory status, the release of cytokines and adipokines from isolated adipocytes was assessed. The results revealed a progressive increase in mean adipocyte size with age in HFD rats when compared to CD rats at each time point; while in CD rats a significant rise in this parameter was found starting at 3 weeks (Figure 4A,G). Levels of IL 6, TNF α, and MCP-1 were significantly and progressively higher in HFD rats than in CD rats with age at every time point. This increase of inflammatory cytokines is more pronounced in CD rats starting about at 12 weeks (Figure 4B–D). Leptin and adiponectin levels augmented progressively in the HFD group compared to controls with age at each time point. In CD rats, these parameters are significantly higher with age from about 12 weeks onward (Figure 4E,F).

### 2.4. Liver Parameters

**Figure 5:**
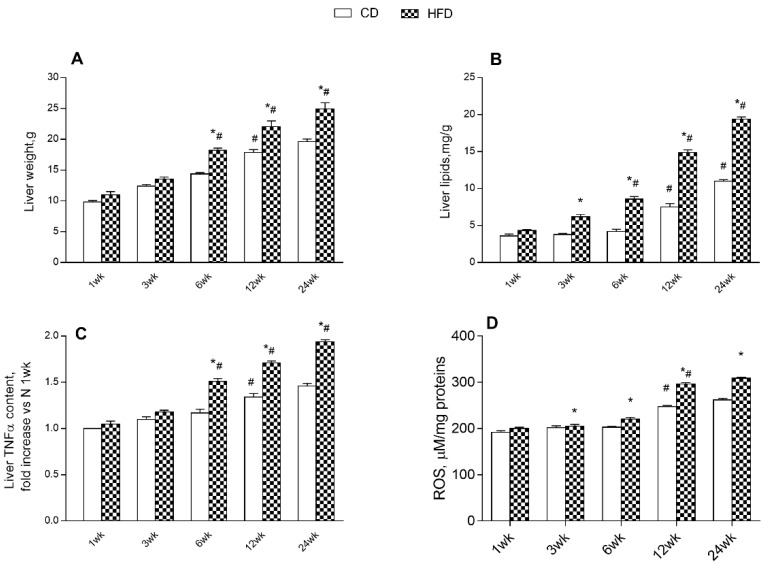
Effects of high-fat diet and aging on liver parameters. All the parameters were measured throughout the experimental period (1–24 weeks): (**A**) liver weight; (**B**) liver lipids, (**C**) tumor necrosis factors-α (TNF-α), and (**D**) ROS content are shown. Data are indicated as means ± SEM from n = 6 animals/group. # Significantly different compared to previous time point, *p* < 0.05; * significantly different compared to CD group, *p* < 0.05.

We evaluated hepatic parameters to analyze the effects of an HFD and aging on liver inflammation, lipid accumulation, and ROS content. The results showed a progressive increase in liver weight and lipid content in HFD and CD rats with age, with higher values in HFD. In the HFD group, these parameters increased significantly with age, starting at 6 weeks of treatment, while increasing in CD rats from 12 weeks (Figure 5A,B). Further, HFD rats exhibited higher hepatic TNF-α and ROS content than the CD group at each time point, with significantly higher values compared to controls from 6 weeks onward (Figure 5C,D). HFD rats exhibited a progressive increase with the age of TNF-α and ROS content, displaying significantly increased values from 6 week and 12 week onward, respectively; while in CD rats an increase in liver TNF-α and ROS content occurred from 12 weeks (Figure 5C,D).

### 2.5. Mitochondrial Function

**Figure 6:**
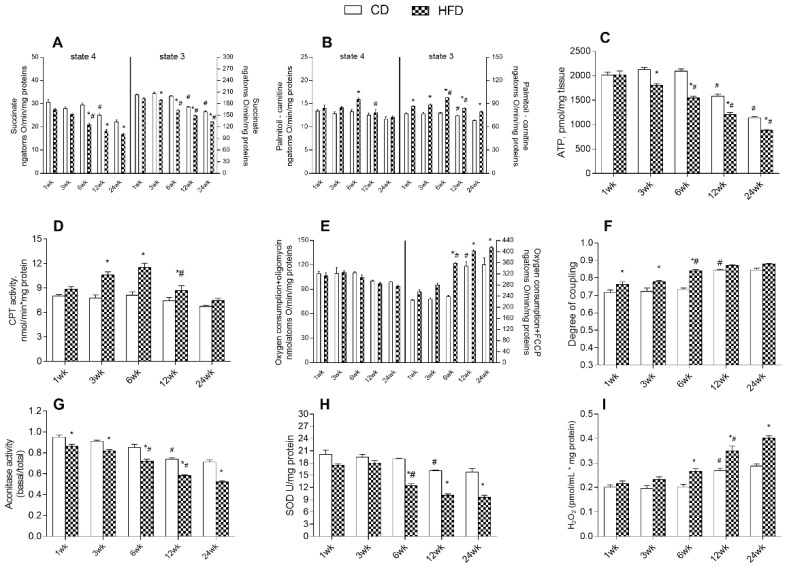
Effects of high-fat diet and aging on mitochondrial respiration parameters in liver. All parameters were measured throughout the experimental period (1–24 weeks): (**A**) Mitochondrial respiration rates measured in the presence of succinate or (**B**) palmitoyl carnitine as substrates; (**C**) ATP content; (**D**) carnitine palmitoyl transferase (CPT) activity; (**E**) oxygen consumption in the presence of oligomycin or uncoupled by carbonyl cyanide 4-(trifluoromethoxy) phenylhydrazone (FCCP); (**F**) degree of coupling; (**G**) aconitase activity; (**H**) superoxide dismutase (SOD) activity and (**I**) hydrogen peroxide (H_2_O_2_) release are shown. Data are indicated as means ± SEM from n = 6 animals/group. # Significantly different compared to previous time point, *p* < 0.05; * significantly different compared to CD group, *p* < 0.05.

Since mitochondria play a crucial role in cellular energy handling, we also investigated the function of these hepatic organelles in the liver. As reported in Figure 6A, mitochondrial oxidative respiratory activity, using succinate as a substrate in the absence of ADP (state 4) showed a significant decrease with age from 6 weeks onward in the HFD, with significantly lower values at 6, 12, and 24 weeks compared to controls. In CD rats, a significant reduction in respiratory rates was observed after 12 weeks of treatment. In presence of ADP (state 3), we observed a progressive decrease in mitochondrial respiratory capacity in both groups of rats, when succinate was used as a substrate. The HFD group exhibited significantly lower values at 3, 6, 12, and 24 weeks than in controls and significantly reduced values with age from 6 weeks of treatment. The CD group showed a respiratory rate (state 3) significantly decreased from 12 weeks onward. When palmitoyl carnitine was used as substrates, in the absence of ADP (state 4), we found a significant increase in respiratory rates at 6 weeks in HFD compared to CD, whereas these values significantly decreased with age from 12 weeks onward. No significant differences occurred in the CD rat group, although a progressive decrease with age was observed. In HFD groups in the presence of ADP (state 3), fatty acids mitochondrial oxidative capacity increased progressively with age up to 6 weeks compared to controls, while a significant reduction occurred after the 6 weeks of treatment. In CD rats a significant reduction was found in state 3 respiration with age starting at 12 weeks (Figure 6B). In the CD group, a significant increase in ATP content was observed starting from the 12th week of treatment. The HFD rats showed a significant decrease in ATP content with age starting from the 6th week, showing significantly lower values than CD rats, starting at the 3rd week (Figure 6C). In addition, carnitine palmitoyl transferase (CPT) activity in the HFD group progressively increased with age up to 6 weeks of treatment but declines significantly after 12 weeks, while no difference was observed in the CD group (Figure 6D). To test mitochondrial efficiency, we measured oxygen consumption rates in the presence of oligomycin, an enzyme inhibitor of ATP synthase, and carbonyl cyanide-p-trifluoromethoxyphenylhydrazone (FCCP), a potent uncoupler of the transmembrane proton gradient. Oligomycin-stimulated respiration showed no difference between HFD and control rats at each time point (Figure 6E). FCCP-stimulated respiration exhibited progressively increased values with age in both groups of rats. The HFD group showed higher values of oxidative consumption at each time point compared to controls, with significantly augmented values starting from 6 weeks onward, while CD rats displayed significantly increased values with age from 12 weeks (Figure 6E). In HFD rats the energy efficiency, assessed as the degree of coupling, increased progressively keeping values higher compared to controls at every time point, with a significant increase observed from the sixth week. In addition, in CD rats, the energy efficiency significantly increased at the 12th week of treatment (Figure 6F). The activity of aconitase and superoxide dismutase (SOD) enzymes decreased progressively in HFD rats with age, showing significantly lower values from 6 weeks onward. Control rats exhibited higher values of aconitase and SOD activity compared to HFD at each time point and a significant decrease with age after 12 weeks (Figure 6G,H). The hydrogen peroxide (H_2_O_2_) yield increased progressively in the HFD group compared to the CD group starting at 6 weeks of treatment. The CD rats showed a significant increase in H_2_O_2_ yield with age from 12 weeks (Figure 6I).

### 2.6. Mitochondrial Function, Dyslipidemia Serum Indicators, Serum and Adipocytes Inflammatory Parameters

**Figure 7:**
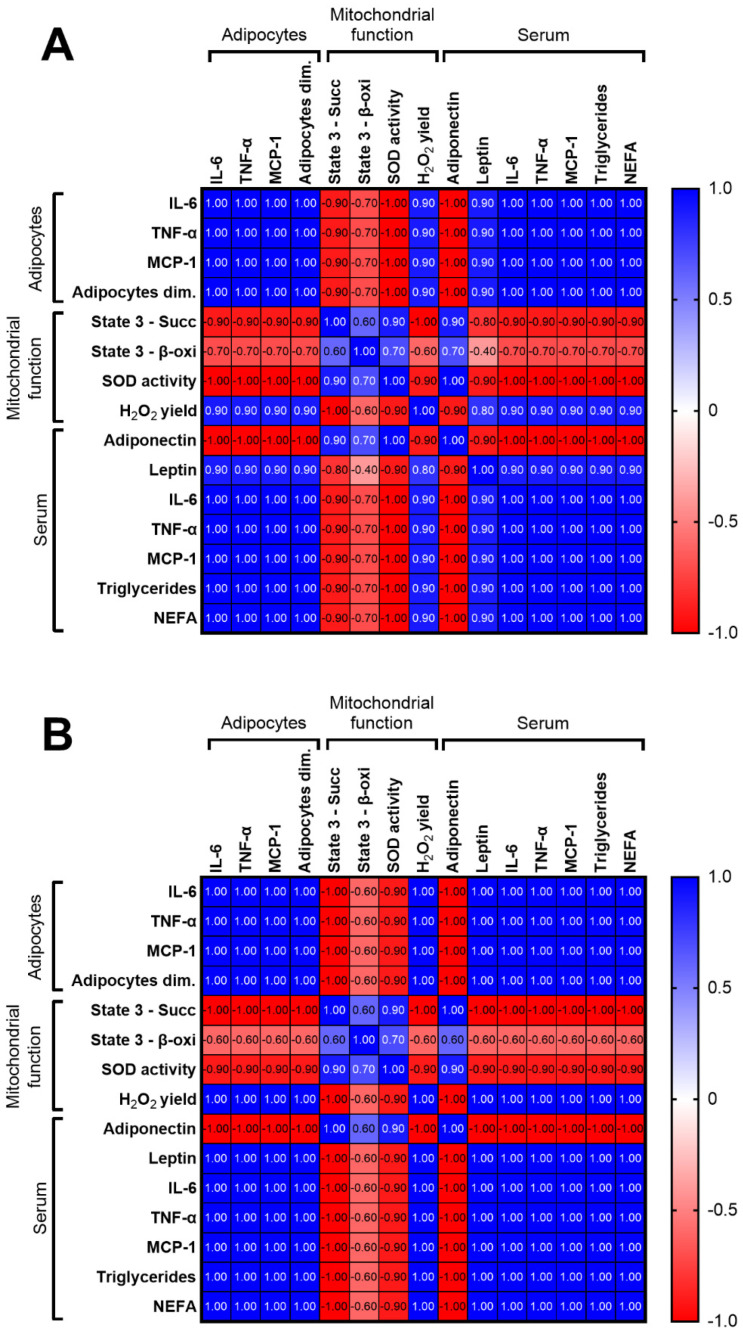
Spearman’s correlation between mitochondrial function parameters, serum dyslipidemia indicators, serum, and adipocytes inflammatory markers in control (**A**) and high-fat diet (**B**) with age.

The relationship between mitochondrial functional parameters, dyslipidemia indicators, serum pro-inflammatory cytokines and inflammatory adipokines release was analyzed. Based on the Spearman correlation analysis, a positive relationship between inflammatory adipokines release, serum inflammatory cytokines, and serum dyslipidemia indicators, both in the control group and in the HFD group with age was found. While, an inverse association between inflammatory serum and adipocyte markers, serum dyslipidemia indicators, and mitochondrial function parameters, was observed more strongly in HFD groups than in CD rats with age (Figure 7A,B). In particular, the best discriminant variable, observed by the above correlation, is the decrease in mitochondrial SOD activity, followed by the ability of mitochondria to oxidize succinate and palmitoyl carnitine, as substrates, in response to the release of pro-inflammatory adipocyte cytokines, such as IL-6, TNF-α, and MCP-1.

## 3. Discussion

Obesity-related imbalance in the fatty acid supply and utilization results mainly in excessive accumulation of intrahepatic lipids and in the development of liver disorders [23]. There is significant evidence to show that aging is an important factor involved in liver fat accumulation, accompanied by abdominal obesity and excessive visceral fat [24]. In the present study, we examined the implication of HFD and aging on changes in body composition, metabolic parameters, and the utilization of liver lipids related to mitochondrial function. We particularly focused on the time course of the obesity-related and aging-related changes in oxidation and storage of liver fatty acids, and on the crosstalk between adipose tissue and liver in the development of inflammatory state and hepatic injury. In agreement with the previous data [25], where we have highlighted that feeding start and the HFD duration are critical in obesity development, the results of this study showed that HFD administration and aging are responsible for body composition changes and metabolic alterations. Indeed, we observed a progressive increase in body fat with age as a consequence of a diminished energy expenditure and increased metabolic efficiency. We also observed alterations in metabolic and inflammatory parameters, such as an increase in markers of dyslipidemia. Serum leptin and adiponectin level alterations, two adipokines correlated with adipose mass [26], were observed mainly in HFD- fed animals. Several studies have proposed that adiponectin levels, besides promoting the oxidation of lipid substrates [27], can be related to the inflammatory state [28]. In fact, its secretion is inhibited by high levels of TNF-α. In particular, the leptin/adiponectin (L/A) ratio negatively correlates with systemic inflammation more closely than adiponectin level. Therefore, the L/A ratio has been suggested as a predictor for low-grade inflammation and the best indicator of metabolic diseases [29]. Our data showed a decrease in adiponectin levels, an increase in L/A ratio, and an increase in TNF-α levels in the HFD group with age, confirming the inverse relation between adiponectin content and inflammatory status. In agreement with our previous data [25], changes in body composition, serum metabolic, and inflammatory parameters started at about 6 weeks of treatment in control rats and progressively increased with age, while in HFD-fed animals, it occurred earlier, at about 3 weeks of treatment, with more pronounced values. An outcome of this study is the strong positive association between body fat and pro-inflammatory adipokine levels with age. Indeed, we found a gradual and proportional rise of adipokine levels with increasing fat mass in rats, underlying a low inflammatory state in HFD and in older CD animals. It is well known that adipocyte size is an important determinant of the secretion of several adipokines [12]. Our results showed a release of adipokines proportional to the average adipocyte size isolated from visceral fat. Furthermore, we observed a progressive increase in the average adipocyte size with age, more pronounced in HFD-fed animals. The increase in adipokine release from adipocytes is most noticeable at about 12 weeks in control rats, although the increase is found as early as 6 weeks, whereas it occurs at about 3 weeks in HFD-fed animals. Although adiponectin serum levels are generally reduced in obesity [30], in vitro its synthesis and release from adipose tissue [31,32] are not reduced. Our results confirm this observation, given the increase in adiponectin release with the observed average size of adipocytes. Several studies have shown that alterations in adipokine production play a role in the pathogenesis of the fatty liver disease [33] and the adipokine profile generated by visceral fat appears particularly harmful [34]. Indeed, the reduced circulating adiponectin levels and increased circulating FFA levels lead to decreased lipid oxidation in non-adipose tissues, thereby triggering the ectopic accumulation of lipids [35,36]. It has also been demonstrated the impact of a long-term high-fat diet feeding on the key features of progressive liver damage [37].

Mitochondrial function has been recognized to playing a key role in the pathogenesis of the liver disease [38,39] and the mitochondrial oxidative capacity has been considered a good predictor of fatty liver diseases [40]. The association between diet-induced ectopic fat storage in the liver and mitochondrial dysfunction is well known [41,42]. Our results revealed that an HFD leads to fatty liver accumulation, alongside impaired mitochondrial function and dysregulated reactive oxygen species (ROS) production, already during the first weeks of treatment, showing a progressive increase with age. Increased mitochondrial ability to oxidize fatty acids, observed in HFD rats up to 6 weeks, might serve as a compensatory mechanism for the continuous and elevated hepatic fatty acid uptake that occurs during the HFD intake, in line with previous results, where we have observed an increase in the lipidic substrates mitochondrial utilization in rats HFD-fed at the 6 week [36]. However, this increase in fatty acid oxidation capacity was insufficient to handle the increased load of hepatic FFA, resulting in lipid accumulation in HFD rat livers. After 6 weeks, we observed a reduction in lipid utilization by liver mitochondria due to the reduction in fatty acid uptake, as observed by lower CPT activity, associated with an impaired mitochondrial respiratory function, as evidenced by diminished state 3 of respiration. All this elicits a further increase in hepatic lipid accumulation after 6 weeks, as shown by the measured lipid content. 

HFD-induced obesity is characterized by low-grade inflammation, metabolic disorders and mitochondrial dysfunctions which are also typical features of aging. In addition, the normal aging process includes the alteration of energy homeostasis, resulting in a gradual increase in adiposity [43]. Mitochondria have been increasingly recognized as important players heavily implicated in aging processes [44]. Our results showed a decrease in hepatic mitochondrial capacity in substrate utilization in control animals, after 12 weeks, underlining a decline in mitochondrial function associated with aging, and a consequent rise in lipid liver accumulation. Moreover, mitochondrial impairment triggered by HFD intake and aging processes contributes to excessive ROS production, as shown by both the high H_2_O_2_ release and ROS content, and the inhibition of aconitase activity. An increase in mitochondrial efficiency, or rather a reduction in mitochondrial uncoupling observed in the HFD group from 6 weeks and in CD rats from 12 weeks, can explain at least in part the increase in ROS production observed in these groups of rats. Indeed, the uncoupling involvement in maintaining mitochondrial membrane potential below the critical threshold for ROS production is well known [45,46]. The increase in ROS production is also confirmed by a relevant decrease in SOD enzyme activity, the first line of defense against oxidative stress [47], especially from 6 weeks onward in HFD rats and from 12 weeks onward in controls. We also found (by Spearman’s correlation analysis) a clear inverse relationship between adipocyte size and inflammatory adipokines with some parameters of liver mitochondrial function, in particular versus the mitochondrial state 3 oxygen consumption rates and the enzymatic antioxidant defenses (SOD activity). This inverse correlation was more evident in HFD than in CD rats. This analysis confirms that the increased release of pro-inflammatory cytokines from adipose tissue impacts the hepatic mitochondrial function. Consequently, this alteration led both to a reduction in energy substrate utilization, responsible for the fat accumulation in the liver, and to a decrease in antioxidant defenses, as indicated by the increased ROS production. These findings indicate a pivotal role played by adipose tissue in liver injury, mediated by mitochondrial function.

In conclusion, our results suggest that HFD and aging would mainly favor the overall development of the dysregulation of adipose tissue function, as revealed by the increase of adiposity, in pro-inflammatory adipokine production and by an increase of dyslipidemia. The increased presence of circulating free fatty acids and lipids, and the compromised secretion/function of the adipocyte hormones, adiponectin and leptin, lead to decreased lipid oxidation in non-adipose tissues, particularly in the liver, causing the ectopic accumulation of lipids. In this scenario, the mitochondrial function impairment induced by an HFD and aging plays a crucial role in decreasing hepatic lipid oxidation, and this can lead to hepatic injury. Thus, the HFD and aging lead to similar metabolic alterations, with different onset times and a common cause: mitochondrial dysfunction. In particular, the HFD anticipates and exacerbates liver mitochondrial dysfunction and metabolic disorders that occur with normal aging processes. 

## 4. Materials and Methods

### 4.1. Materials Reagent

All chemicals were purchased from Sigma-Aldrich (St. Louis, MO, USA), unless otherwise specified.

### 4.2. Animal Diet

Young male Wistar rats at 60 days of age and 345.7 g of average body weight were individually caged in a temperature-controlled room and exposed to a daily 12/12 h light/dark cycle with free access to chow diet and drinking water. Rats were divided into two experimental groups according to a different dietary regimen: the first group (control diet, CD) received a standard diet (10.6% fat J/J, 29% protein J/J, 60.4% carbohydrate J/J); the second group (HFD) received high-fat diet (40% fat J/J, 29% protein J/J, 31% carbohydrate J/J). The animals from both groups were fed with the respective diet for 1, 3, 6, 12, or 24 weeks (n = 6 for each group and time point). An additional group (n = 6) was sacrificed at the beginning of the study to establish baseline measurements of body compositions. At the end of the experimental treatments, the rats were anesthetized by i.p. injection of chloral hydrate (40 mg/100 g body weight), decapitated with a guillotine, and the blood was taken from the inferior cava vein. Serum or plasma was obtained by centrifugation at 1000× *g* for 10 min at 4 °C. The liver and adipose epididymal tissues were quickly excised and transferred in the appropriate buffer to isolate the mitochondria from the liver and the adipocytes from the visceral tissue. All the samples that were not immediately used were stored at −80 °C. Procedures involving animals and their care were conducted in conformity with international and national laws and policies (EU Directive2010/63/EU for animal experiments, ARRIVE guidelines, and the Basel declaration including the 3R concept). The procedures reported here were approved by the Institutional Committee on the Ethics of Animal Experiments (CSV) of the University of Naples Federico II (Permit Number: 2010/0149862) and by the Ministero della Salute.

### 4.3. Body Composition and Energy Balance

During treatments, body weight and food intake were monitored daily to obtain body weight gain and gross energy intake. Energy balance assessments were conducted during the 24 weeks of feeding by comparative carcass evaluation [48]. The gross energy density for CD or HFD (15.8 or 21.9 kJ/g, respectively) and the energy density of the feces and the carcasses were determined by bomb calorimetric (Parr adiabatic calorimetric; Parr Instrument Company, Moline, IL, USA), as previously reported [25,49]. In detail, for the comparative body energy calculation, the carcasses were weighed, autoclaved for 90 min, chopped into small pieces, thoroughly mixed, and homogenized with a mass of water equal to twice the carcass weight in a Polytron homogenizer (Polytron Kinematica AG, Littau/Lucerne, Switzerland). Aliquots of the homogenates were desiccated at 70 °C in a vacuum oven. Small pellets (about 200 mg) of the dried homogenate were made and burned under constant volume by a calorimetric bomb. The amount of heat released in the reaction can be calculated using the equation q = −CΔT, where C is the heat capacity of the calorimeter and ΔT is the temperature change. The obtained values were normalized for gram of sample and converted into kJ units. ME intake was determined by subtracting the energy measured in feces and urine from the gross energy intake, which was determined from the daily food consumption and gross energy density. Evaluation of the energy, fat, and protein content in animal carcasses was conducted according to a published protocol [48]. In detail, the body’s lipid content was estimated by using the Folch method [50]. Energy efficiency was calculated as the percentage of body energy gain per ME intake, and energy expenditure was determined as the difference between ME intake and body energy gain. Body energy gain was calculated as the difference between the body energy content at the end of the treatment and the energy content of the rats sacrificed at the beginning of the experiment (baseline measurements).

### 4.4. Serum Parameters

The serum levels of TG, NEFA, and cholesterol were measured by the colorimetric enzymatic method using commercial kits (SGM Italia, Rome, Italy, and Randox Laboratories Limited, Crumlin, UK). The serum levels of IL-1b, IL-6, TNF-α, MCP-1 (BioVendor, Brno, Czechia), adiponectin, and leptin (B-Bridge International, Mountain View, CA, USA) were measured using commercially available ELISA kits. 

### 4.5. Isolation Adipocytes and Adipokines

Animals were sacrificed, and epididymal fat pads were removed and stored in DMEM containing 20 µg/mL gentamicin. Connective tissue and visible blood vessels were removed with scissors. For isolation of adipocytes, the adipose tissues were minced and digested in Krebs-Ringer-phosphate buffer (KRP; 154 mM NaCl, 100 mM NaH_2_PO_4_, 154 mM KCl, 154 mM MgSO_4_, 110 mM CaCl_2_, pH 7.4) containing 100 U/mL of collagenase and BSA (4% *w*/*v*) for 60 min at 37 °C in shaking water bath. After this step, the undigested tissue was removed by filtration through a nylon mesh with a pore size of 250 µm (VWR, Darmstadt, Germany). The floating adipocytes were washed three times with KRP containing BSA (0.1% *w*/*v*) and were incubated in DMEM culture medium (10%FBS, 1X Pen/Strep). Representative fields were photographed using an inverted microscope Leica DMi1 (Leica Microsystems, Wetzlar, Germany). After 24 h the media were immediately stored at −80 °C for subsequent analysis. Macrophage contamination was excluded by microscopic analysis.

### 4.6. Measurement of Adipokines Release

The concentrations of adiponectin and leptin in the cell culture media were measured using highly sensitive sandwich ELISAs (B-Bridge International, Mountain View, CA, USA). The concentrations of IL-6, TNF-α, and MCP-1 were measured using a highly sensitive sandwich ELISA (BioVendor, Brno, Czechia) [51]. The amount of proteins in the media from the cellular incubations was normalized for 10^6^ cells.

### 4.7. Hepatic Lipid Content

Lipid content was determined according to the method described by Folch [50]. Briefly, liver was weighted, chopped into small pieces, thoroughly mixed, and upon the addition of 2 parts of water (*w*/*w*), it was homogenized by a Polytron homogenizer. Homogenate aliquots were weighted, and the lipid content was determined gravimetrically after extraction in chloroform–methanol and evaporation by a rotating evaporator (Heidolph, Schwabach, Germany), the lipid amount was finally expressed as mg/g of tissue.

### 4.8. Hepatic ROS Assay

ROS levels were measured as previously reported [52]. An aliquot of tissue homogenate was diluted in 100 mM potassium phosphate buffer (pH 7.4) and incubated in a final concentration of 5 mM dichlorofluorescein diacetate (Sigma-Aldrich) in dimethyl sulfoxide for 15 min at 37 °C. The dye-loaded samples were centrifuged at 12,500× *g* per 10 min at 4 °C. The pellet was resuspended in 5 mL of 100 mM potassium phosphate buffer (pH 7.4) at 4 °C, and incubated for 60 min at 37 °C. The fluorescence measurements were performed at 488 nm for excitation and 525 nm for emission wavelengths. ROS was quantified using a dichlorofluorescein standard curve in dimethyl sulfoxide (0–1 mM).

### 4.9. Mitochondria Preparation and Analysis

Liver aliquots were finely minced and washed in a medium containing 100 mM KCl, 50 mM Tris-HCl, pH 7.5, 5 mM MgCl_2_, 1 mM EDTA, 5 mM EGTA, 0.1% (*w*/*v*) fatty acid-free bovine serum albumin (BSA). Tissue fragments were homogenized with the above medium (1:8, *w*/*v*) in a Potter Elvehjem homogenizer (Heidolph, Kelheim, Germany) set at 500 rpm (4 strokes/min) and filtered through sterile gauze. The homogenate was then centrifuged at 1000× *g* for 10 min, and the resulting supernatant was again centrifuged at 3000× *g* for 10 min. The mitochondrial pellet was washed twice and finally resuspended in a medium containing 80 mM LiCl, 50 mM HEPES, 5 mM Tris-PO_4_, 1 mM EGTA and 0.1% (*w*/*v*) fatty-acid-free BSA, pH 7.0. The protein content of the mitochondrial suspension was determined through Bradford colorimetric test (Biorad) using BSA as standard.

Mitochondrial oxygen consumption was polarographically measured by a Clark-type electrode (Yellow Springs Instruments, Yellow Springs, OH, USA) at 30 °C. In detail, isolated mitochondria (0,5 mg protein/ml) were incubated in a medium containing 30 mM KCl, 6 mM MgCl_2_, 75 mM sucrose, 1 mM EDTA, 20 mM KH_2_PO_4_ pH 7.0, and 0.1% (*w*/*v*) fatty acid-free bovine-serum albumin (BSA). In the presence of 10 mM succinate, 3.75 μM rotenone and 0.6 mM ADP, the oxygen consumption was measured. The rate of mitochondrial fatty acid oxidation was assessed in the presence of malate (2.5 mM), palmitoyl-L-carnitine (40 µM) and ADP (0.6 mM). State 3 oxygen consumption was measured in the presence of ADP, while state 4 was obtained in the absence of ADP. High quality of mitochondrial preparations was indicated by high respiratory control ratio (RCR) values in all groups, calculated as the ratio between the states 3 and 4, according to Estabrook [53]. In addition, in control experiments, we assured the quality of our mitochondrial preparation by checking that the contamination of the mitochondria by other ATPase-containing membranes was lower than 10%, and the addition of cytochrome c (3 nmol/mg protein) only enhanced the state 3 respiratory rate by approximately 10% [54].

The degree of coupling was determined in the liver by applying the equation by Cairns et al. (1998) [55]:

$$\mathrm{degree}\;\mathrm{of}\;\mathrm{coupling}=\sqrt{1-(Jo)sh/(Jo)\;\;\;unc}$$

where (Jo)sh represents the oxygen consumption rate (OCR) in the presence of oligomycin that inhibits ATP synthase, and (Jo)unc is the uncoupled rate of oxygen consumption induced by FCCP, which dissipates the transmitochondrial proton gradient. (Jo)sh and (Jo)unc were measured as above using succinate (10 mmol/L) and rotenone (3.75 mmol/L) in the presence of oligomycin (2 mg/ml) or FCCP (1 mmol/L), respectively. 

CPT activity was followed spectrophotometrically as CoA-sH production by the use of 5,5’-dithiobis (nitrobenzoic acid) (DTNB) and palmitoyl-Coa 10 μM as substrate. The medium consisted of 50 mM KCl, 10 mM Hepes (pH 7.4), 0.025% Triton X-100, 0.3 mM DTNB, and 10–100 pg of mitochondrial protein in a final volume of 1.0 ml. The reaction was followed at 412 nm at 25 °C in a thermostated spectrophotometer and the enzymatic activity was calculated from: E412 = 13,600/ (M × cm) [56]. The rate of mitochondrial H_2_O_2_ release was assayed by following the linear increase in fluorescence (excitation 312 nm and emission 420 nm) due to the oxidation of homovanillic acid in the presence of horseradish peroxidase [57]. SOD specific activity was measured in a medium containing 0.1 mM EDTA, 2 mM KCN, 50 mM KH_2_PO_4_, pH 7.8, 20 mm cytochrome c, 5 mm xanthine, and 0.01 U of xanthine oxidase. Enzyme activity was measured spectrophotometrically (550 nm) at 25 °C, by monitoring the decrease in the reduction rate of cytochrome c by superoxide radicals, generated by the xanthine–xanthine oxidase system. One unit of SOD activity is defined as the concentration of enzyme that inhibits the cytochrome c reduction of 50% in the presence of xanthine and xanthine oxidase [58,59]. Aconitase activity in the liver was carried out in a medium containing 30 mM of sodium citrate, 0.6 mM MnCl_2_, 0.2 mM NADP, 50 mM TRIS-HCl pH 7.4, and 2 units of isocitrate dehydrogenase. The formation of NADPH was followed spectrophotometrically (340 nm) at 25 °C. The level of aconitase activity measured equals active aconitase (basal level). Aconitase inhibited by ROS in vivo was reactivated so that the total activity could be measured by incubating mitochondrial extracts in a medium containing 50 mM dithiothreitol, 0.2 mM Na_2_S, and 0.2 mM ferrous ammonium sulphate [60,61]. ATP content in the liver was measured using a commercially available kit (Abcam, ab83355), as previously reported [62].

### 4.10. Statistical Analysis

All data are presented as means SEM. Differences among groups were compared by unpaired *t*-test. Differences were considered statistically significant at *p* < 0.05. The # symbol was used to indicate the significant differences vs the immediately preceding time point value, the symbol * indicates, at each time point, the significant differences vs the control group. All analyses were performed using GraphPad Prism (GraphPad Software, San Diego, CA, USA). Spearman correlation analysis was carried out for some parameters for every pair of Y data set (correlation matrix), two-tailed *p* value (confidence interval 95%).

## Data Availability

Data is contained within this article.
